# How maladaptive internet use translates into online learning disengagement: the mediating role of academic anxiety and the moderating role of self-handicapping among Chinese adolescents

**DOI:** 10.3389/fpsyg.2026.1894026

**Published:** 2026-08-05

**Authors:** Wenbo Deng, Hao Zhang, Zhijing Zhao, Haoyan Huang

**Affiliations:** 1 School of Journalism and Communication, Yangzhou University, Yangzhou, China; 2 School of Education, Yangzhou University, Yangzhou, China; 3 Faculty of Educational Sciences, University of Helsinki, Helsinki, Finland

**Keywords:** academic anxiety, maladaptive internet use, online education, online learning disengagement, self-handicapping

## Abstract

**Introduction:**

While online education provides flexible learning opportunities, it also exposes students to risks of maladaptive Internet use andpsychological distress.

**Methods:**

This study investigated the mechanisms linking maladaptive Internet use to online learning disengagement by examining the mediating role of academic anxiety and the moderating role of self-handicapping within a moderated mediation model, drawing on social cognitive theory, self-worth theory, and compensatory Internet use theory. A total of 404 10th-grade Chinese high school students (M_age = 15.47 years, 48.3% female) participated in the self-report surveys.

**Results:**

Results indicated that (1) maladaptive Internet use had a positive association with online learning disengagement both directly and indirectly through its association with academic anxiety; (2) Self-handicapping negatively moderated boththe paths from maladaptive Internet use and academic anxiety to disengagement, attenuating the strength of these associations with disengagement. All students exhibited greater disengagement as maladaptive Internet use or anxiety increased,yet the magnitude of this increase was roughly twice as large among students low in self-handicapping (*β* = 0.47 and 0.39) than among those high in self-handicapping (*β* = 0.19 and 0.20), revealing differential sensitivity to risk factors; (3) self-handicapping did not moderate the path frommaladaptive Internet use to academic anxiety

**Discussion:**

Findings extend self-worth theory by showing that self-handicapping is associated with a comparatively weaker behavioral response to maladaptive Internet use and academic anxiety, consistent with a short-term attributional buffering function. They also suggest that creating stable, low-pressure learning environments and providing emotional support may help sustain engagement, particularly among students low in self-handicapping.

## 1 Introduction

Accelerated by the COVID-19 pandemic, the global expansion of online education has reshaped contemporary teaching and learning, providing flexible and accessible learning opportunities while presenting substantial challenges to student self-regulation and academic adaptation (Majadly et al., 2024; Global MOOC and Online Education Alliance, 2025). Although digital platforms support continuous learning, the blurred boundaries between study and leisure, together with reduced in-person supervision, have increased the prevalence of maladaptive Internet use among students (Takahashi et al., 2018; Rahmani et al., 2024). Extensive digital engagement often disrupts learning routines, increases mind wandering, and fosters dysfunctional online behaviors that exacerbate academic anxiety and learning disengagement (Jung and Lee, 2018; Bergdahl, 2022; Deng et al., 2022; Metin-Orta and Demirtepe-Saygılı, 2023).

Despite growing research on online learning engagement, the mechanisms about how maladaptive Internet use contributes to online learning disengagement, and which factors can buffer its contributions, remain largely unexplored (Greenhow et al., 2022; Hamilton et al., 2023; Panigrahi et al., 2018; Tate and Warschauer, 2022). Given the increasing popularization of online education and serious drawbacks of online learning disengagement, this investigation is imperative to understand how to prevent and compensate for this issue.

To address these gaps, the present study examined the association of maladaptive Internet use with online learning disengagement, and further explored the mediating role of academic anxiety and moderating role of self-handicapping to uncover the underlying dynamics. Students from Chinese senior high school were surveyed, due to their comparatively high vulnerability. Specifically, Chinese students usually adapt to controlling learning environment with rigorous external regulation, and have insufficient opportunities to develop self-regulatory skills (Zhang et al., 2018; Chen et al., 2025). Therefore, it may be difficult for many Chinese students to balance online learning with digital distractions, leading to excessive, uncontrolled Internet use that interferes with academic functioning (Zhu et al., 2022; CNNIC, 2023).

### 1.1 Maladaptive internet use and online learning disengagement

Social Cognitive Theory suggests that learning is not merely an individual cognitive activity but a socially embedded process (Bandura, 2001). In this framework, students’ learning behaviors and outcomes are deeply shaped through triadic social interactions that provide models of effective learning, feedback on performance, vicarious reinforcement, social comparison standards, and regulatory support (Schunk and DiBenedetto, 2020; Zimmerman, 1989). These processes help students regulate learning behaviors, develop positive academic emotions, and have a desirable learning engagement (Schunk and DiBenedetto, 2020; Zimmerman, 1989). Yet, the social interaction opportunities are largely insufficient during online learning, which is associated with an increased tendency toward maladaptive Internet use, which in turn is linked to learning disengagement (Miao et al., 2022; Richardson et al., 2017).

Prior studies have defined online learning disengagement as a persistent state in which students withdraw from, or fail to actively invest in, online learning activities (Bergdahl, 2022). As a critical indicator of maladaptive learning state, it reflects insufficient cognitive, behavioral, affective, and social involvement in learning tasks, characterized by inattention, avoidance of learning requirements, lack of emotional investment, and low persistence in online learning environments (Hoi and Hang, 2021; Bong et al., 2024). This maladaptive state has profound negative impacts on students’ cognitive functioning, academic performance, and emotional health (Volungis et al., 2020; Sunday et al., 2021; Skulmowski and Xu, 2022), raising increasing concerns for understanding its causes for possible prevention.

While online learning disengagement is influenced by a complex set of individual and environmental factors, maladaptive Internet use emerges as one of the most influential, conceptualized as compulsive, excessive, or uncontrolled online behavior that disrupts daily responsibilities and impairs academic focus (Caplan, 2010). Due to insufficient social interactions and supervision, online learning raises a high demand for students’ self-regulation and persistent of efforts, which may overwhelm their immature cognitive capabilities (Wang et al., 2022; Wu et al., 2023). Consequently, many students suffer from maladaptive Internet use during online learning, wasting considerable time and cognitive resources and finally contributing to online learning disengagement (Özhan and Kocadere, 2019; Martin and Borup, 2022; Hutain and Michinov, 2024). Accordingly, we propose: *H1*: Maladaptive Internet Use is positively associated with online learning disengagement.

### 1.2 The mediating role of academic anxiety

To further understand the underlying mechanism of online learning disengagement due to maladaptive Internet use, this study further surveyed the role of academic anxiety, defined as students’ cognitive, physiological, and behavioral anxious responses triggered by educational environments and academic tasks (Cassady, 2022). On the one hand, compensatory Internet use theory (Kardefelt-Winther, 2014) suggests that maladaptive Internet use can, in turn, elevate academic anxiety. Students who turn to digital devices to escape real-world stressors are more likely to experience heightened social disconnection, and this disconnection usually intensifies students’ academic anxiety (Cleveland-Innes and Campbell, 2012; Li et al., 2023). Meanwhile,the substantial amount of time and energy devoted to excessive online activities reduces students’ learning efficiency, leaving insufficient time for academic tasks and further intensifying their academic anxiety (Jin et al., 2024). Consistent with compensatory Internet use theory’s broader emphasis on a self-reinforcing cycle between psychological distress and escapist Internet use, we conceptualize maladaptive Internet use and academic anxiety as mutually reinforcing over time, rather than assuming a single, unidirectional sequence running only from Internet use to anxiety.

On the other hand, academic anxiety is a representative academic emotion in shaping students’ learning adaptation, with a particularly strong influence in online settings than in traditional face-to-face classrooms (Cheng et al., 2023). Cognitively, it consumes attentional and working memory resources that students would otherwise devote to learning tasks (Eysenck et al., 2007); motivationally, it triggers avoidance-oriented coping, prompting students to withdraw from challenging tasks or seek psychological refuge in non-academic online activities (Fernandes et al., 2025). Both pathways converge on reduced behavioral engagement and weakened persistence. From the perspective of self-worth theory (Covington, 1992), this motivational withdrawal can also be understood as a self-protective response: by disengaging rather than exerting full effort and risking failure, students shield their self-worth from being directly implicated by poor academic performance. Moreover, compared with traditional classrooms, online learning environments amplify these effects because students have to self-monitor their progress without the immediate cues of teacher presence and peer behavior, in which academic anxiety is less likely to be regulated through external support. As a consequence, recent evidence shows that anxiety negatively predicts online learning engagement (Wang et al., 2022; Wu et al., 2023). These findings have identified academic anxiety as a crucial mechanism linking environmental stressors to disengagement in digital learning contexts. Therefore, we hypothesize that academic anxiety may mediate the association between maladaptive Internet use and online learning disengagement (H2).

### 1.3 The moderating role of self-handicapping

While maladaptive Internet use and academic anxiety may exacerbate online learning disengagement, some studies have found inconsistent results (e.g., Romo et al., 2025; Yu et al., 2020), suggesting potential moderators to buffer their negative impacts. This study particularly focused on the moderating role of self-handicapping, known as an anticipatory defensive strategy where individuals intentionally create obstacles to their own performance (Urdan and Midgley, 2001). Grounded in self-worth theory, self-handicapping allows students to externalize potential failure (attributing it to external factors) while internalizing success (crediting it to their own ability), thereby protecting their self-esteem from the stigma of perceived incompetence (Jones and Berglas, 1978). This self-protective strategy may be especially likely to emerge, where social supervision and evaluation are largely absent, make students more inclined to engage in self-impeding behaviors (Török et al., 2018).

Although self-handicapping results in some negative learning outcomes including disengagement (Martin et al., 2001; Schwinger et al., 2014), it may weaken rather than eliminate, the strength of the deteriorative paths from maladaptive Internet use to academic anxiety and online learning disengagement, without resolving their underlying negative long-term consequences. Specifically, by attributing potential learning setbacks to external obstacles (e.g., distraction by the Internet) rather than to lack of ability, self-handicapping may reduce the extent to which maladaptive Internet use-related difficulties are internalized as personal failure, temporarily helping students maintain a positive self-image; however, this self-protective function operates primarily in the short term and does not eliminate the underlying negative association with learning engagement, nor does it offset the well-documented long-term costs of self-handicapping, such as reduced academic achievement and diminished self-efficacy (Covington, 1992; Rappo et al., 2017). For instance, by reframing anxiety-inducing experiences as controllable and externally caused, students may temporarily preserve a sense of competence and remain marginally engaged with learning tasks under emotional distress, even though this strategy carries longer-term motivational costs (Schwinger et al., 2014; Boruchovitch et al., 2022).

Despite these potential short-term self-protective function, rare empirical studies have examined the moderating role of self-handicapping. This gap is particularly important in online learning settings, where reduced supervision and weakened social cues amplify both academic anxiety and the temptation to escape into non-academic Internet use (Wu et al., 2023). Taken together, social cognitive theory, self-worth theory, and compensatory Internet use theory jointly inform the present model: social cognitive theory motivates the broader link between online learning conditions and maladaptive Internet use and disengagement; compensatory Internet use theory explains why maladaptive Internet use elevates academic anxiety; and self-worth theory explains both why academic anxiety promotes disengagement and why self-handicapping, as a self-worth-protective strategy, may attenuate without eliminating these associations. Based on the implications from prior studies, we hypothesize: *H3*: Self-handicapping moderates the relationship between maladaptive Internet use and online learning disengagement. *H4*: Self-handicapping moderates the relationship between academic anxiety and online learning disengagement. *H5*: Self-handicapping moderates the relationship between maladaptive Internet use and academic anxiety.

### 1.4 The present study

**Figure 1:**
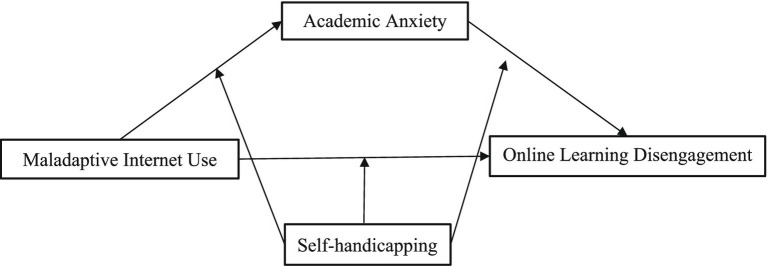
Hypothesized moderated mediation model linking maladaptive internet use, academic anxiety, self-handicapping, and online learning disengagement.

In sum, this study aims to investigate (a) whether maladaptive Internet use is positively associated with online learning disengagement among Chinese senior high school students, (b) whether academic anxiety mediates this association, and (c) whether self-handicapping moderates the associations among maladaptive Internet use, academic anxiety and online learning disengagement. Drawing on self-worth theory (Covington, 1992) and social cognitive theory (Bandura, 1986), the proposed moderated mediation framework conceptualizes online learning disengagement as the joint outcome of dysfunctional digital coping (maladaptive Internet use), emotional distress (academic anxiety), and self-protective motivation (self-handicapping). The six hypotheses derived in Sections 1.1–1.3 are summarized in Figure 1.

## 2 Methods

### 2.1 Participants and procedure

This study is a cross-sectional survey conducted to capture data on the online learning status of senior high school students. After approval from the academic committee and consent from the guardians, the questionnaire was published on the school’s online platform. Participants were asked to complete a self-report questionnaire online that explicitly emphasized the anonymity of personal information and the participant’s right to withdraw from the study at any time. Convenience sampling was used in this study. Data were collected during the period of nationwide school closures and full-time online learning prompted by the COVID-19 pandemic in China. In total, 450 students from two high schools in a provincial capital city in China participated in the study. After the exclusion of incomplete or invalid responses, 417 valid questionnaires were initially obtained. Following data screening, 13 cases were further excluded: one with an invalid age code (999) and 12 with missing-code responses on the self-handicapping items, yielding a final analytic sample of 404 senior high school students. Participants were aged between 14 and 17 years (M = 15.47 years, SD = 0.65), comprising 209 males (51.7%) and 195 females (48.3%).

### 2.2 Measures

The research team employed a standardized translation and back-translation procedure to adapt three questionnaires into Chinese, with modifications to the wording to better align with the context of high school online learning. Subsequently, the team sought feedback from two psychology experts holding doctoral degrees regarding the translated and revised questionnaires. Based on the expert recommendations, the questionnaire items were further refined to finalize the content. Confirmatory factor analyses (CFAs) and reliability analyses, reported below for each scale, were used to verify the psychometric quality of the adapted instruments.

#### 2.2.1 Online learning disengagement

Online learning disengagement was measured by reverse-coding the Online Learning Engagement Scale developed by Hoi and Hang (2021). Consistent with common practice in the engagement literature, where engagement and disengagement are treated as opposite poles of a single behavioral continuum rather than categorically distinct constructs, low scores on the original engagement items (reflecting reduced effort, participation, and persistence) were used to index disengagement. This scale originally assesses four dimensions of engagement: behavioral, emotional, cognitive, and social engagement. All items were rated on a five-point Likert scale ranging from 1 (Strongly Disagree) to 5 (Strongly Agree). To align with the conceptual framework of this study, which focuses on academic withdrawal, all items were reverse-coded (e.g., a score of 1 was converted to 5) so that higher scores consistently represented a higher level of online learning disengagement. CFA indicated good model fit: *χ*^2^/df = 3.81, CFI = 0.95, TLI = 0.93, RMSEA = 0.08, SRMR = 0.04; Cronbach’s *α* = 0.95.

#### 2.2.2 Academic anxiety

The Online Academic Anxiety scale used selected questions from the Classroom-Related Anxiety Questionnaire (Pekrun et al., 2002). The final instrument was revised to be appropriate for online learning situations. It contained 7 items rated on a five-point scale (1 = strongly disagree, 5 = strongly agree). CFA indicated acceptable fit (*χ*^2^/df = 15, *p* < 0.001, RMSEA = 0.18, SRMR = 0.05, CFI = 0.93), and Cronbach’s α = 0.93.

#### 2.2.3 Academic self-handicapping

The self-handicapping scale was adopted from a scale developed by Huang (2006) and re-validated (Bingbing et al., 2022). The instrument consists of 14 items across two dimensions: behavioral academic self-handicapping (T1, T3, T5, T7, T11, T13, and T14) and claimed academic self-handicapping (T2, T4, T6, T8, T9, T10, T12). Items were rated on a five-point scale (1 = strongly disagree, 5 = strongly agree). CFA indicated acceptable fit (*χ*^2^/df = 8.34, *p* < 0.001, RMSEA = 0.07, SRMR = 0.04, CFI = 0.91), and Cronbach’s *α* = 0.97.

#### 2.2.4 Maladaptive internet use

Maladaptive Internet Use was assessed using an adapted version of the Mobile Phone Dependence Scale (MPDS), which is based on the 10-item Smartphone Addiction Scale-Short Version (SAS-SV; Kwon et al., 2013). Because maladaptive Internet use was measured using an adapted mobile phone/smartphone dependence scale, this construct captures students’ general problematic Internet/smartphone use tendencies rather than Internet use specific to online class sessions or Internet use that directly interferes with learning tasks. To ensure the instrument precisely captured the pathological nature of online engagement rather than general device usage, minor linguistic modifications were made to the original items. For instance, phrases referring to general smartphone use were refined to emphasize compulsive online activities and digital preoccupation (e.g., changing “using smartphone” to “using internet”). This adaptation aligns the scale with the theoretical constructs of Maladaptive Internet Use, focusing on the dysfunctional behaviors that interfere with students’ academic focus.

CFA indicated acceptable fit (*χ*^2^/df = 7.14, *p* < 0.001, CFI = 0.94, RMSEA = 0.07, SRMR = 0.04), and Cronbach’s α = 0.94. While the RMSEA value is slightly above the traditional threshold, the combination of high incremental fit indices (CFI) and a low SRMR confirms that the adapted scale remains a valid and reliable measure for this adolescent sample.

### 2.3 Analysis strategy

Prior to analysis, two data-cleaning steps were performed: cases with an invalid age code (999) were removed (*n* = 1), and the value −2 in the Self-Handicapping items was treated as a missing code rather than a valid response; cases missing on any Self-Handicapping item were excluded listwise (*n* = 12). The final analytic sample comprised 404 students. Gender and age were included as control variables in all analyses. All analyses were performed using SPSS 26.0 with the PROCESS macro v4.1 (Hayes, 2013). First, Harman’s single-factor test and Confirmatory Factor Analysis (Mplus 8.3) were used to examine common method bias and the factorial structure of all instruments. Second, descriptive statistics and Pearson correlations were computed for all study variables, with gender treated as a control variable based on prior evidence of gender differences in academic anxiety and self-handicapping (Chaplin and Aldao, 2013; Yu and McLellan, 2019). Third, all continuous predictors were mean-centered prior to creating interaction terms, following Hayes (2013) recommendation. The moderated mediation model was tested using PROCESS Model 59, in which maladaptive Internet use was the predictor (X), academic anxiety the mediator (M), online learning disengagement the outcome (Y), and self-handicapping the moderator (W) on all three paths (X → M, X → Y, M → Y). Bootstrap resampling with 5,000 samples was used to estimate confidence intervals for direct, indirect, and conditional effects. Conditional effects were probed at low (M − 1 SD), medium (M), and high (M + 1 SD) levels of self-handicapping, and simple slopes analyses were conducted to interpret significant interactions. The Index of Moderated Mediation was also computed to assess whether the indirect effect of maladaptive Internet use through academic anxiety on disengagement varied with self-handicapping.

## 3 Results

### 3.1 Common method Bias

To address common method bias, the anonymity of participants’ responses was emphasized, and Harman’s single-factor test (Podsakoff et al., 2003) was conducted. Fourteen factors emerged with eigenvalues greater than 1, and the first factor accounted for only 35.63% of the total variance, below the 40% threshold indicating no serious common method bias.

### 3.2 Descriptive statistics and bivariate correlations

Prior research has consistently demonstrated the significant influence of gender on academic anxiety and self-handicapping behaviors (Chaplin and Aldao, 2013; Yu and McLellan, 2019). Accordingly, gender was included as a control variable throughout subsequent analyses. Table 1 presents the means, standard deviations, skewness, kurtosis, and correlation coefficients for the key study variables. Skewness and kurtosis values supported the assumption of approximate normality.

**Table 1:** Descriptive statistics and bivariate correlations (*N* = 404).

Variable	M	SD	Skew	Kurt	1	2	3	4	5	6
Gender	0.48	0.50	0.07	−2.00	1.00					
Age	15.47	0.65	0.42	0.18	1.00					
3. Maladaptive internet use	21.89	10.42	0.68	−0.27	−0.01	−0.15^**^				
4. Academic anxiety	14.78	7.33	0.71	−0.34	−0.09	−0.09	0.54^***^	1.00		
5. Self-handicapping	25.05	12.83	1.51	2.12	0.12^**^	−0.08	0.46^***^	0.37^***^	1.00	
6. Online learning disengagement	30.06	12.02	0.82	0.85	0.03	−0.04	0.41^***^	0.41^***^	0.16^**^	1.00

*N* = 404. Gender (0 = male, 1 = female) was treated as a control variable in subsequent regression analyses. * *p* < 0.05, ** *p* < 0.01, *** *p* < 0.001.

The bivariate correlation results (Table 1) indicated significant relationships among all study variables in the expected directions. Maladaptive Internet use was positively associated with both academic anxiety (*r* = 0.54, *p* < 0.001) and self-handicapping (*r* = 0.46, *p* < 0.001). Online learning disengagement exhibited significant positive correlations with academic anxiety (*r* = 0.41, *p* < 0.001), self-handicapping (*r* = 0.16, *p* < 0.01), and maladaptive Internet use (*r* = 0.41, *p* < 0.001). Self-handicapping was also positively related to academic anxiety (*r* = 0.37, *p* < 0.001). These patterns provide preliminary support for the hypothesized model.

### 3.3 Moderated mediation model

Table 2 presents the regression results of the moderated mediation model. For the mediator model, both Maladaptive Internet Use (*β* = 0.48, *p* < 0.001) and self-handicapping (*β* = 0.19, *p* < 0.001) have positive associations with academic anxiety, although their interaction was non-significant (*β* = −0.06, *p* = 0.076). Together, the predictors accounted for 32.9% of the variance in academic anxiety, *F*(5, 398) = 39.06, *p* < 0.001. For the dependent variable model, maladaptive Internet use (*β* = 0.33, *p* < 0.001) and academic anxiety (*β* = 0.30, *p* < 0.001) were significantly associated with online learning disengagement, whereas self-handicapping had no direct main effect (*β* = −0.01, *p* = 0.797). Crucially, the two interaction terms involving self-handicapping were both significant: maladaptive Internet use × self-handicapping (*β* = −0.14, *p* < 0.001) and academic anxiety × self-handicapping (*β* = −0.10, *p* = 0.019), supporting Hypotheses 4 and 5. The model explained 28.7% of the variance in online learning disengagement, *F*(7, 396) = 22.74, *p* < 0.001.

**Table 2:** Moderated mediation model (PROCESS model 59)—updated coefficients.

Predictor	b	SE	*β*	*t*	*p*
Outcome A: academic anxiety (*R*^2^ = 0.329, *F*(5, 396) = 39.06, *p* < 0.001)					
Gender	−1.52	0.61	−0.10	−2.50	0.013*
Age	−0.07	0.47	−0.01	−0.14	0.886
Maladaptive internet use (X)	0.34	0.03	0.48	10.08	< 0.001***
Self handicapping (W)	0.11	0.03	0.19	3.96	< 0.001***
X × W	−0.003	0.002	−0.06	−1.78	0.076
Outcome B: online learning disengagement (*R*^2^ = 0.287, *F*(7, 396) = 22.74, *p* < 0.001)					
Gender	2.08	1.05	0.09	1.99	0.047*
Age	0.13	0.80	0.01	0.17	0.869
Maladaptive internet use (X)	0.38	0.06	0.33	5.91	< 0.001***
Academic anxiety (M)	0.49	0.09	0.30	5.55	< 0.001***
Self handicapping (W)	−0.01	0.05	−0.01	−0.26	0.797
X × W	−0.013	0.003	−0.14	−3.76	< 0.001***
M × W	−0.012	0.005	−0.10	−2.36	0.019*

Coefficients b are unstandardized; *β* are fully standardized. All continuous predictors were mean-centered prior to creating interaction terms. Coefficients with magnitudes less than 0.01 are presented with three decimal places to avoid rounding to zero. *N* = 404. * *p* < 0.05, *** *p* < 0.001.

**Figure 2:**
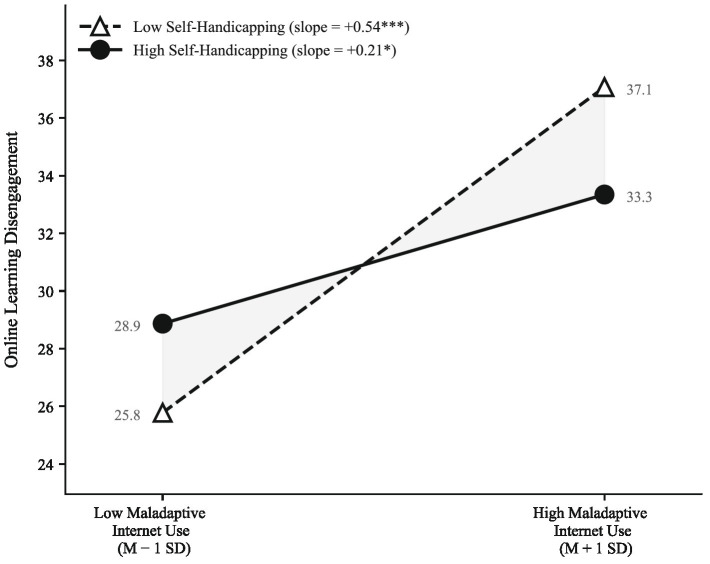
Simple slopes of the relationship between maladaptive internet use and online learning disengagement at low (*M* − 1 *SD*) and high (*M* + 1 *SD*) levels of self-handicapping.

**Figure 3:**
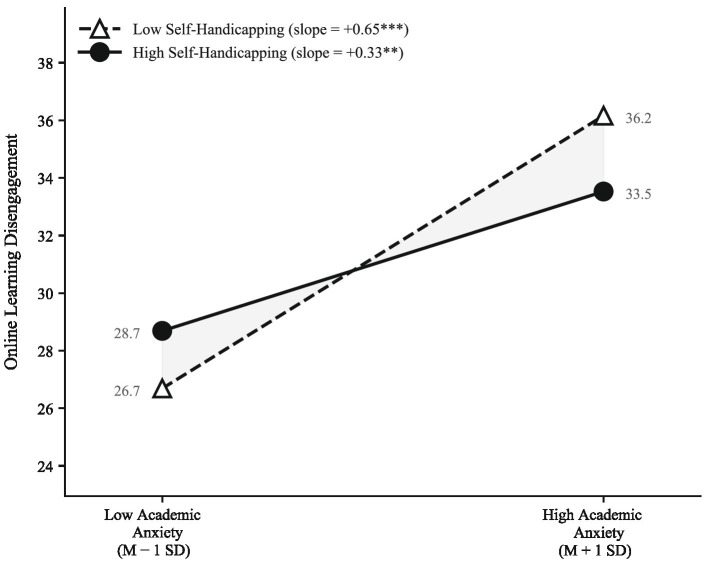
Simple slopes of the relationship between academic anxiety and online learning disengagement at low and high levels of self-handicapping. Both panels share an identical Y-axis range to facilitate direct visual comparison.

Then, conditional direct and indirect effects were estimated at three levels of self-handicapping: low (M − 1 SD), medium (M), and high (M + 1 SD). As shown in Table 3 and Figures 2,3, three patterns emerged. First, the conditional direct effect of Maladaptive Internet Use on online learning disengagement was positive at all three levels but decreased monotonically as self-handicapping increased, from b = 0.54 [95% Boot CI (0.33, 0.78)] at low Self Handicapping, to b = 0.38 [95% Boot CI (0.21, 0.55)] at the mean, and to *b* = 0.22 [95% Boot CI (0.02, 0.40)] at high Self Handicapping. All three effects were significant. Second, the conditional direct effect of academic anxiety on disengagement followed an identical pattern, with the slope decreasing from *b* = 0.65 [95% Boot CI (0.32, 0.95)] at low Self Handicapping, to b = 0.49 [95% Boot CI (0.26, 0.70)] at the mean, to b = 0.33 [95% Boot CI (0.10, 0.58)] at high Self Handicapping. Third, the conditional indirect effect of Maladaptive Internet Use on disengagement through academic anxiety was significant across all levels but attenuated with increasing self-handicapping: *b* = 0.24 [95% Boot CI (0.11, 0.39)] at low Self Handicapping, *b* = 0.16 [95% Boot CI (0.08, 0.26)] at the mean, and b = 0.10 [95% Boot CI (0.03, 0.19)] at high Self Handicapping. The Index of Moderated Mediation was −0.005 [95% Boot CI (−0.011, 0.000)], at the boundary of statistical significance. Together, these results indicate that self-handicapping attenuated, but did not reverse, the positive effects of Maladaptive Internet Use and academic anxiety on online learning disengagement, supporting H4 and H5.

**Table 3:** Conditional direct, indirect, and total effects.

Pathway/level of moderator	Effect	Boot SE	95% Boot CI
Conditional direct effect: maladaptive internet use → disengagement			
Low self handicapping (W = M − 1 SD)	0.54	0.12	[0.326, 0.780]
Mean self handicapping (W = M)	0.38	0.09	[0.214, 0.554]
High self handicapping (W = M + 1 SD)	0.22	0.10	[0.015, 0.398]
Conditional direct effect: academic anxiety → disengagement			
Low self handicapping (W = M − 1 SD)	0.65	0.16	[0.324, 0.946]
Mean self handicapping (W = M)	0.49	0.11	[0.262, 0.702]
High self handicapping (W = M + 1 SD)	0.33	0.12	[0.096, 0.581]
Conditional indirect effect: maladaptive internet use → anxiety → disengagement			
Low self handicapping (W = M − 1 SD)	0.24	0.07	[0.113, 0.385]
Mean self handicapping (W = M)	0.16	0.04	[0.082, 0.255]
High self handicapping (W = M + 1 SD)	0.10	0.04	[0.025, 0.194]
Index of moderated mediation	−0.005	0.003	[−0.011, 0.000]

Effects are estimated at low (M − 1 SD), mean, and high (M + 1 SD) levels of Self-Handicapping. Bootstrap CIs based on 5,000 resamples. All conditional direct and indirect effects are positive and statistically significant; their magnitudes attenuate as Self-Handicapping increases.

## 4 Discussion

During the rapid expansion of online education, the joint roles of dysfunctional digital behavior, emotional distress, and self-protective motivation in student disengagement has remained insufficiently understood. This study aimed to elucidate the mechanisms underlying the association between maladaptive Internet use and online learning disengagement, by examining the mediating role of academic anxiety and the moderating role of self-handicapping. Was directly and indirectly associated with online learning disengagement via academic anxiety, while self-handicapping attenuated the strength of these associations. Specifically, students with low self-handicapping showed roughly twice the behavioral response to elevated maladaptive Internet use and academic anxiety than students high in self-handicapping, illuminating self-handicapping’s role as an attributional buffer that dampens behavioral disengagement under distress.

### 4.1 Maladaptive internet use, academic anxiety, and online learning disengagement

Consistent with hypotheses 1 and 2, this study found that maladaptive Internet use had a positive association with online learning disengagement (H1), both directly and indirectly through academic anxiety (H2). These findings align with a growing body of evidence highlighting the detrimental impact of maladaptive digital behaviors and emotional distress on digital academic persistence. Specifically, excessive and unregulated Internet use has been associated with emotional maladjustment, especially increased academic anxiety, by disrupting daily routines, reducing face-to-face social interaction, and exposing individuals to information overload (Phanphech et al., 2022). In online learning environments, this association may be more pronounced, due to the lack of social support to buffer it (Faulconer and Griffith, 2022; Hilliard et al., 2020), which may in turn be linked to reduced cognitive and behavioral engagement and higher levels of withdrawal. Together, these findings are consistent with emotional distress playing a role in the association between maladaptive Internet use and online disengagement.

Notably, after accounting for academic anxiety, the direct path from maladaptive Internet use to online learning disengagement remained statistically significant, implying a further dynamic behind emotional distress process. For instance, maladaptive Internet use can displace academic time, reduce concentration, and foster a habit of digital distraction (Tao et al., 2024), which requires further explorations to comprehensively understand its dynamics.

### 4.2 Self-handicapping as a buffer: differential sensitivity to risk factors

While maladaptive Internet use and academic anxiety had positive associations with online learning disengagement, the strength of these associations was negatively moderated by self-handicapping, confirming hypotheses 3 and 4. This finding suggests that self-handicapping is associated with a weaker behavioral response to maladaptive Internet use and academic anxiety in online learning. This attenuated association, however, should not be interpreted as evidence that self-handicapping is generally beneficial or adaptive; rather, it more plausibly reflects the fact that students high in self-handicapping already display a relatively stable, avoidance-oriented pattern of behavior, which leaves comparatively less additional room for disengagement to increase as risk factors rise.

This pattern is consistent with Self-Worth Theory (Covington, 1992), in which students who habitually engage in self-handicapping behaviors, such as excuse-making, procrastination, and recourse to external barriers, possess an attributional buffer that insulates self-worth from the impact of academic difficulties. It is important to emphasize, however, that this same attributional repertoire is also well documented to carry substantial long-term costs, including lower academic achievement, reduced self-efficacy, and heightened risk of learned helplessness (e.g., Schwinger and Stiensmeier-Pelster, 2011; Zuckerman and Tsai, 2005). The present pattern should therefore be understood as reflecting a short-term, self-protective function rather than a generally adaptive strategy. When maladaptive Internet use or anxiety rises among these students, the resulting distress may be less likely to translate into further disengagement, given their existing defensive repertoire (Ferradás et al., 2019). In contrast, students low in self-handicapping lack this attributional cushion, having fewer cognitive resources to dissociate distress from self-evaluation, so their disengagement more closely tracks fluctuations in these stressors (Schwinger and Stiensmeier-Pelster, 2011). Importantly, this asymmetry does not imply that low self-handicapping is maladaptive. Under low-risk conditions (low maladaptive Internet use and low anxiety), low-self-handicapping students actually showed the lowest predicted level of disengagement (see Figures 2,3, left points). The asymmetry simply means that low-self-handicapping students are more responsive to their psychological environment, whether benign or threatening. They benefit more from low-risk conditions but show greater disengagement under high-risk conditions.

It is also worth noting that the index of moderated dediation was at the boundary of statistical significance. Although the conditional indirect effect of maladaptive Internet use through academic anxiety was clearly weaker among high-self-handicapping students than low-self-handicapping students, the contrast between these two conditional indirect effects, taken as a whole, did not reliably exclude zero. The moderation of the second stage of the mediation pathway (from academic anxiety to online learning disengagement) is therefore more clearly established than the moderation of the indirect effect as an aggregate quantity, in which self-handicapping primarily moderates the immediate behavioral response to academic anxiety, rather than systematically altering the holistic paths of “maladaptive Internet use →Academic anxiety → disengagement” chain.

Additionally, contrary to hypothesis 5, self-handicapping did not significantly moderate the effects of maladaptive Internet use on academic anxiety pathway. This finding indicates that the affective pathway from problematic digital behavior to emotional distress reflects a direct and consistent stress response. Greater maladaptive Internet use is consistently associated with higher academic anxiety, regardless of self-handicapping levels. From a theoretical perspective, this clarifies the boundary conditions of self-handicapping. It primarily moderates behavioral and motivational outcomes such as disengagement, where self-worth concerns are most salient (Martin et al., 2001; Schwinger et al., 2022), but exerts less influence on affective reactions to environmental stress (Zuckerman et al., 1998). In other words, self-handicapping shapes how students behave under distress, that is, whether they withdraw from learning, but not whether they feel distress in the first place. By specifying which links in the moderated mediation chain are buffered by self-handicapping (the behavioral output) and which are not (the affective input), the present results refine the boundary conditions of Self-Worth Theory in the digital learning context.

### 4.3 Implications

Although these data were collected during the unique context of pandemic-induced full-time online learning, the identified mechanisms, namely the joint role of maladaptive Internet use, academic anxiety, and self-handicapping in shaping disengagement, likely apply more broadly to any context in which students depend heavily on autonomous online learning, including post-pandemic hybrid models, MOOCs, and emergency remote teaching scenarios. These findings have several practical implications. First, *strengthening emotional support and psychological safety* should be prioritized: given the direct link between academic anxiety and disengagement (*b* = +0.49, *p* < 0.001), educators should provide timely positive feedback and one-on-one communication, especially for students low in self-handicapping whose behavioral response to distress is roughly twice as strong as that of their high self-handicapping peers. Second, scaffolded feedback and social interaction should be embedded in instructional design. By providing clear learning milestones, peer support groups, and collaborative projects, educators can redirect students’ urge to seek “digital refuge” toward meaningful engagement with learning tasks. Third, *digital agency, not mere restriction*, should guide maladaptive Internet use interventions: given the strong link between maladaptive Internet use and academic anxiety (*b* = 0.34, *p* < 0.001), focus modes, time-management training, and emotion-regulation skills (e.g., mindfulness, brief physical activity) help students distinguish purposeful internet use from anxiety-driven escape surfing. Finally, *strengthening protective factors for low self-handicapping students* is essential. The present findings indicate that students low in self-handicapping are more sensitive to both risk and protective factors in their learning environment. While they are more vulnerable when maladaptive Internet use or academic anxiety rises, they also benefit more from low-distress conditions. Educators and parents should therefore prioritize creating a stable, low-pressure online learning environment for these students — for example, predictable schedules, immediate access to teacher support, and frequent low-stakes feedback. Because of their heightened responsiveness, even modest improvements in environmental quality may yield disproportionately large reductions in disengagement.

### 4.4 Limitations and future directions

Several limitations warrant consideration. First, the cross-sectional design precludes causal inference. Because compensatory Internet use theory frames escapist online behavior and psychological distress as mutually reinforcing rather than one-way, this design cannot eliminate the possibility that academic anxiety triggered problematic Internet use instead of the reverse. Future research should employ longitudinal or experimental designs to track these dynamic relationships over an extended period and disentangle the causal direction of the reinforcing cycle. Second, the study relies solely on self-report data and may be subject to social desirability and common method bias; multi-informant or behavioral data (e.g., learning management system logs) would strengthen future work. Third, the sample is limited to Chinese senior high school students limiting the generalizability of the findings to other cultural, educational, and age groups. Fourth, the academic anxiety scale showed a relatively high RMSEA (0.18), suggesting room for refinement of the adapted instrument. Finally, data were collected during the COVID-19 lockdown when online learning was both mandatory and the sole mode of instruction. Levels of maladaptive Internet use and disengagement in our sample may reflect the heightened stress and reduced autonomy of that period; replication in voluntary or hybrid learning contexts is warranted.

## 5 Conclusion

By examining the joint dynamics of dysfunctional digital behavior, emotional distress, and self-protective motivation in relation to online learning disengagement, this study uncovered a moderated mediation model among their associations. Specifically, maladaptive Internet use is associated with online learning disengagement both directly and indirectly through academic anxiety, and self-handicapping negatively moderates both of these associations with disengagement but not the path from maladaptive Internet use to academic anxiety. These findings extend self-worth theory by showing that self-handicapping is associated with a comparatively weaker behavioral response to distress-related disengagement. Consistent with the interpretation offered in Section 4.2, however, this pattern is better understood as reflecting an already-stable, avoidance-oriented behavioral repertoire among high-self-handicapping students, together with the well-documented long-term maladaptive consequences of self-handicapping (e.g., reduced achievement, diminished self-efficacy), rather than as evidence of a generally adaptive buffering function. The findings further extend compensatory Internet use theory by showing that this attenuating role does not extend to the affective pathway linking maladaptive Internet use to academic anxiety, which remained consistent regardless of self-handicapping level. Practically, fostering psychological safety, scaffolded feedback, and stable low-pressure environments may be more effective for sustaining engagement in online learning than efforts focused solely on discouraging self-handicapping; nonetheless, given its documented long-term costs, helping students develop healthier, non-avoidant coping strategies remains an important goal, particularly for supporting the more environmentally responsive group of students low in self-handicapping, who stand to benefit most from such supportive conditions.

## Data Availability

The raw data supporting the conclusions of this article will be made available by the authors, without undue reservation.
